# Effective treatment of refractory, locally metastatic squamous cell carcinoma of the leg with isolated limb perfusion: a case report with unexpected long progression-free interval

**DOI:** 10.3389/fonc.2025.1549683

**Published:** 2025-03-21

**Authors:** Danai-Dionysia Kanatoula, Sebastian A. Wohlfeil, Jens Jakob, Peter Hohenberger, Jochen Utikal

**Affiliations:** ^1^ Department of Dermatology, Venereology, and Allergology, University Medical Center and Medical Faculty Mannheim, Heidelberg University, and Center of Excellence in Dermatology, Mannheim, Germany; ^2^ Skin Cancer Unit, German Cancer Research Center (DKFZ), Heidelberg, Germany; ^3^ Skin Cancer Unit, Dermato-Oncology, German Cancer Research Center (DKFZ) Hector Cancer Institute at the University Medical Center Mannheim, Mannheim, Germany; ^4^ Division of Surgical Oncology, Department of Surgery, Mannheim University Medical Center, University of Heidelberg, Mannheim, Germany

**Keywords:** SCC, squamous cell carcinoma, treatment, isolated limb perfusion, rhTNFα

## Abstract

Non-melanoma skin cancer (NMSC) is one of the most commonly diagnosed human malignancies and its incidence is steadily increasing. Locally advanced cutaneous squamous cell carcinoma of the extremities that is refractory to standard therapies can be challenging to treat, with amputation of the limb being the ultima ratio treatment. Here we present a 67-year-old female patient with metastatic SCC of the leg refractory to standard therapies who was effectively treated with isolated limb perfusion and is free of any sign of relapse since more than 3 years. This case report provides a brief review of the recent literature on isolated limb perfusion and how this effective treatment can preserve the patient’s quality of life by avoiding radical surgery and its negative consequences through limb salvage.

## Introduction

Non-melanoma skin cancers (NMSC) are among the most commonly diagnosed malignancies in humans, especially in the Caucasian population. NMSC is the most common cancer in Europe, Australia and New Zealand among men and women and in the United States among men, with the incidence steadily increasing (1, 2). When cutaneous squamous cell carcinoma (cSCC) presents as a localized lesion, microscopically controlled excision is the standard treatment. In locally advanced cases, radiotherapy, electrochemotherapy or systemic therapy with anti-PD1 antibodies, EGFR inhibitors or chemotherapeutic agents may be used. If the above-mentioned therapies fail, amputation can be performed as a last option.

Hyperthermic isolated limb perfusion (ILP) is a proven and registered therapy to avoid amputation in soft tissue sarcomas (3). In ILP, the extremity is isolated from the body circulation by a tourniquet, the femoral artery and vein are cannulated and connected to a heart-lung machine combined with a blood heating device to maintain extremity circulation and enable treatment with recombinant human tumor necrosis factor alpha (rhTNF, Beromun™) and chemotherapeutic agents like melphalan or cisplatinum at high concentrations. The aim is to destroy cancer cells locally in a single session and to minimize systemic side effects. This case report demonstrates how ILP can achieve an unexpectedly long and unique progression-free interval in a patient with refractory locally metastatic squamous cell carcinoma of the leg.

## Case description

A 67-year-old Caucasian female patient was diagnosed with cSCC with a tumor thickness of 3.5 mm on the anterior edge of the right tibia in August 2019 in another hospital. Apart from Bowen’s disease on the right lower leg, which had been excised with clear margins four months before, no other epithelial skin tumors were known in the patient’s personal or family history. The patient denied long-term sun exposure. The patient was not immunocompromised and had no other relevant comorbidities, except for arterial hypertension and hypothyroidism, which were treated with candesartan 8mg, torasemide 10mg and L-thyroxine 150µg, each taken once daily in the morning. In August 2019, a wide excision of the tumor was performed. Until March 2020, several recurrences of the cSCC, including satellite metastases were diagnosed and treated surgically. As cutaneous filiae on the right tibia progressed, local radiotherapy (03-04/2020) and electrochemotherapy (07/2020) were administered without sufficient improvement. Since local recurrences progressed, immunotherapy with an anti-PD-1 inhibitor, cemiplimab 350 mg every 3 weeks, was indicated and started in 10/2020. The patient developed a lichen planus in 04/2021, and treatment was discontinued. Besides this, the immune-related adverse event made it even more difficult to assess progress or regression of cSCC.

In May 2021, the patient presented to our skin cancer center for the first time and her case was discussed in our interdisciplinary tumor board. With further local progression, an isolated hyperthermic extremity perfusion with rhTNF-alpha, melphalan, and cisplatinum was recommended and performed in July 2021.

Preoperative cardiac echocardiography and pulmonary function tests were performed. During the procedure, the patient was placed under general anesthesia. The thigh was isolated using a tourniquet, and systemic heparinization was administered with 8,000 International Units (IU) of heparin. Catheters were inserted into the iliac artery and vein, and an extracorporeal circuit was established. The limb was perfused at a maximum flow of 400ml with a perfusion pressure of 140mmHg. The initial temperature in the gastrocnemius muscle was 32°C. After 30 minutes, once the temperature reached 36°C, 1mg of rhTNF-alpha, 80mg of melphalan, and 10mg of cisplatinum were administered at 5-minute intervals. After 60 minutes, the limb was gradually returned to normal temperature, and circulation was restored.

**Figure 1:**
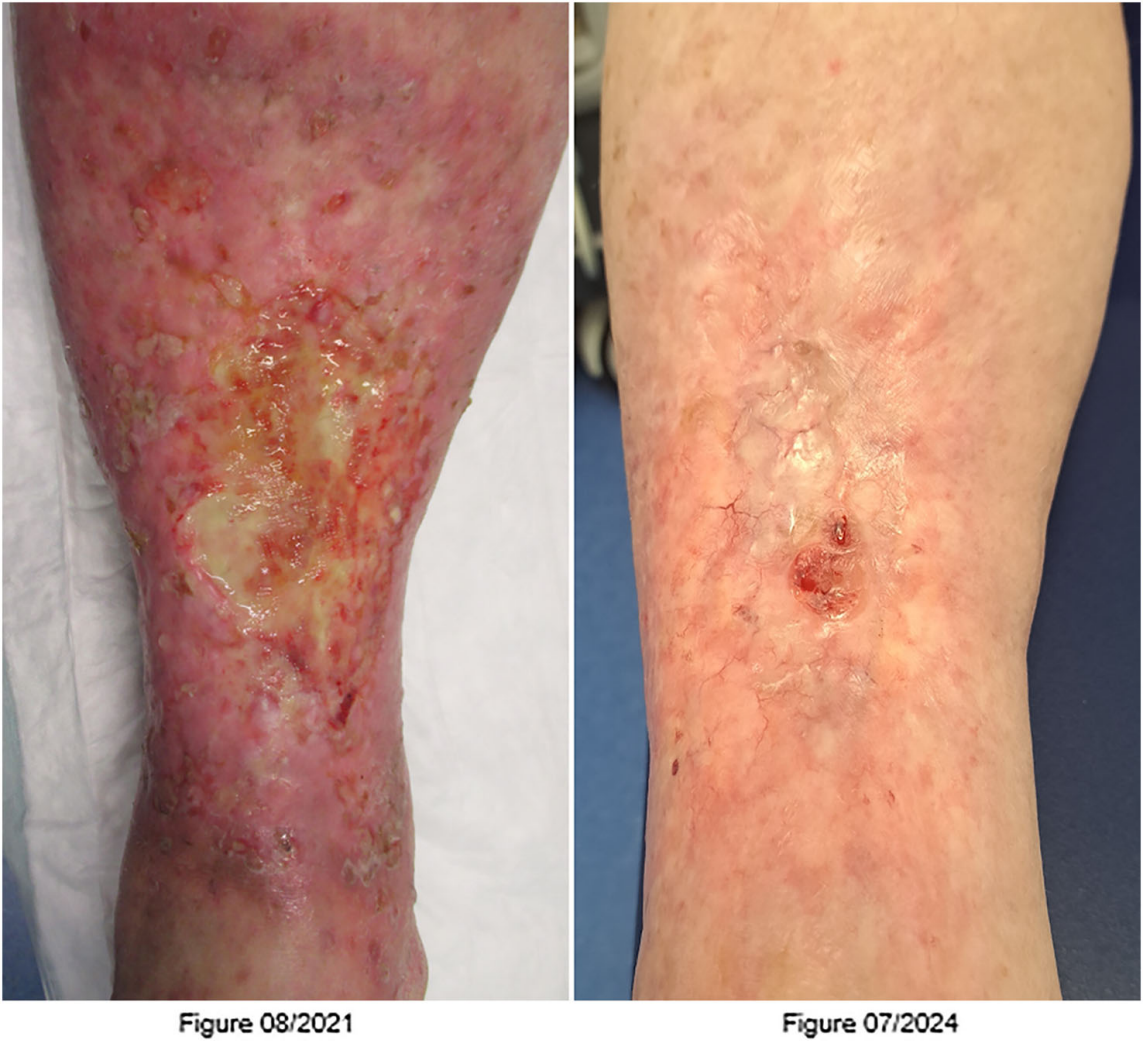
Figure 08/2021: Lower leg with post-ILP dermatitis 1 month after limb perfusion. Figure 07/2024: Lower leg with complete regression of the cutaneous filiae and isolated tension blisters.

The patient developed grade III toxicity (considerable erythema and edema +/- blistering) typically for the combination of hyperthermia, and cisplatinum (**Figure 1**) (4). She was hospitalized and treated with topical corticosteroids and disinfectants. Besides, sterile puncture of blisters was performed. At the first re-staging, the patient showed a complete response with complete regression of the skin metastases. To date, the patient remains free from tumor, which continues to be confirmed by close follow-up.

## Discussion

Isolated hyperthermic limb perfusion is a form of regional chemotherapy that was first introduced clinically in 1957 at Tulane University by Creech et al. for the treatment of advanced limb tumors (5). Until the 1990s, only cytotoxic agents at high concentrations were used to treat limb threatening tumors such as in-transit metastasized melanoma or locally advanced sarcoma (6). With the introduction of rhTNFα which has distinct effects on the tumor microenvironment and tumor vasculature in the immediate (e.g. with increased tumor vascular permeability and increased chemo-uptake) and late (e.g. apoptosis of tumor vasculature endothelium) phase of the treatment, ILP became much more effective (6). Indeed, limb preservation in locally advanced sarcoma may be achieved in up to 80% of all amputation candidates by applying TNF-ILP and is a registered treatment in Europe.

In ILP, the limb circulation is isolated from the body circulation to allow high dosages of cytostatic drugs, however more importantly, enables the application of rhTNFα which is used at a dose of 1 to 3mg which accounts for the 50-100fold of the maximum tolerated dose. For this purpose, a heart-lung machine with a heating device is connected to the limb to perfuse the limb, regulate tissue temperature and supply the limb with oxygen. The standard drugs used are the cytostatic agent melphalan and the cytokine rhTNF-α, which potentiates the uptake of melphalan into the tumor tissue (7).

TNF-ILP has two distinct risks for the patient: First, leakage of rhTNF to the systemic circulation during the procedure despite application of a tourniquet. TNF leakage may lead to severe and immediate systemic inflammatory response syndrome (SIRS) which may be life threatening if not immediately treated. The drug Beromun™ (Belpharma, Luxemburg) is only supplied to accredited specialized cancer centers with experience in extremity tumor treatment and proven standard operating procedures and expertise in TNF-ILP. Second, the combination of rhTNF and cytotoxic drugs may lead to local toxicity including compartment syndrome (grade IV according to Wieberdink). The complications that occur after isolated hyperthermic limb perfusion were classified by Wieberdink in 1982 and are the following: toxicity grade I (no reaction), grade II (slight erythema and edema), grade III (considerable erythema and edema +/- blistering), grade IV (threatened or actual compartment syndrome), grade V (requiring amputation) (4).

The patient survived the operation well. Postoperatively, she developed grade III toxic dermatitis with severe pain requiring in-patient treatment with local glucocorticoids and antiseptic compresses as well as systemic analgesics. The dermatitis healed without complications. After several months of frustrating treatment attempts with standard therapies, the patient felt relieved and completely satisfied with the overall result after the ILP.

The main indications are therapy-refractory in-transit metastases of malignant melanoma and advanced soft tissue sarcomas that do not respond to conventional therapies. Case reports and smaller studies also report promising response rates of ILP in other tumors such Merkel cell carcinoma (MCC), desmoid tumors or non-hodkin lymphoma (8–11).21/12/2024 15:27:00.

In a case series of 12 patients with in-transit metastases of Merkel cell carcinoma treated with isolated extremity perfusion and extremity infusion, 11 patients showed complete remission (CR) and 1 patient showed partial remission (PR) (9). Local metastases recurred in 4 patients after 22 months, although amputation was avoided in all patients (9).

In 25 patients with desmoid tumors who were treated with ILP, a response rate of 72% was achieved after 84 months (10). 16 patients showed a PR and 2 patients a CR (10). Amputation was avoided in all but 3 patients.

In 30 patients with locally advanced cutaneous squamous cell carcinoma treated with ILP with melphalan and TNF-α, a response rate of 81% was achieved after 25 months and complete remission (CR, 59%) was achieved in 16 patients (8). 7 patients developed local recurrences after an average of 9 months and 5 patients showed disease progression (PD). The 2-year survival rate was 67% and amputation was avoided in 80% of patients (8).

In 15 patients with locally advanced skin tumors (12 with SCC and 3 with MCC) who were treated with ILP with TNF-α, melphalan and interferon, a CR was achieved in 60% (9 patients) and a PR in 27% (4 patients) (12). 2 patients (13%) did not respond to treatment. Amputation was avoided in 80% (12 patients) and local recurrence occurred in 4 patients (27%) (12).

A systematic review of 22 studies involving 2,018 ILPs in patients with unresectable locally advanced melanoma of the limbs revealed a median complete response rate of 58.20% and a median overall response rate of 90.35% (13). The median complete response rate for ILP with melphalan plus TNF was 68.90%, while the median complete response rate for ILP with melphalan was 46.50% (13). The median five-year overall survival rate was 36.50%, with a median overall survival interval of 36.7 months (13). The Wieberdink IV and V regional toxicity rates were 2% and 0.65%, respectively (13).

A strength of this case report is that it presents a unique and unexpected case of refractory locally metastatic squamous cell carcinoma with a long progression-free interval after ILP. This case report recommends ILP as an alternative treatment to avoid amputation in similar cases when all standard treatments have failed. A major limitation is its poor generalizability, as it is not clear which patient and tumor characteristics lead to a good response to the ILP. Further research is needed to answer these questions.

## Conclusion

An amputation can not only have an enormous physical and psychological impact on the patient’s quality of life but can also massively restrict patient’s functionality. For this reason, ILP should be considered as an alternative treatment option for locally advanced, refractory skin tumors. Although ILP does not prolong overall survival, it is an effective therapy for local tumor control, preventing amputation and improving patients’ quality of life by preserving their limbs.

## Data Availability

The original contributions presented in the study are included in the article/supplementary material. Further inquiries can be directed to the corresponding author.
